# The Influence of COVID-19 on the Well-Being of People: Big Data Methods for Capturing the Well-Being of Working Adults and Protective Factors Nationwide

**DOI:** 10.3389/fpsyg.2021.681091

**Published:** 2021-06-21

**Authors:** Xiyang Zhang, Yu Wang, Hanjia Lyu, Yipeng Zhang, Yubao Liu, Jiebo Luo

**Affiliations:** ^1^Department of Psychology, University of Akron, Akron, OH, United States; ^2^Department of Political Science, University of Rochester, Rochester, NY, United States; ^3^Goergen Institute for Data Science, University of Rochester, Rochester, NY, United States; ^4^Department of Computer Science, University of Rochester, Rochester, NY, United States

**Keywords:** COVID-19, big data, personality, social connectedness, positive affect and negative affect

## Abstract

The COVID-19 outbreak has affected the lives of people across the globe. To investigate the mental impact of COVID-19 and to respond to the call of researchers for the use of unobtrusive and intensive measurement in capturing time-sensitive psychological concepts (e.g., affect), we used big data methods to investigate the impact of COVID-19 by analyzing 348,933 tweets that people posted from April 1, 2020 to April 24, 2020. The dataset covers 2,231 working adults, who are from 454 counties across 48 states in the United States. In this study, we theorize the similarity and dissimilarity between COVID-19 and other common stressors. Similar to other stressors, pandemic severity negatively influenced the well-being of people by increasing negative affect. However, we did not find an influence of pandemic severity on the positive affect of the people. Dissimilar to other stressors, the protective factors for people during COVID-19 are not common factors that make people resilient to stress and they echo the unique experience during COVID-19. Moreover, we analyzed the text content of 348,933 tweets through Linguistic Inquiry Word Count (LIWC) and word cloud analysis to further reveal the psychological impact of COVID-19 and why the protective factors make people resilient to the mental impact of COVID-19. These exploratory analyses revealed the specific emotions that people experienced and the topics that people are concerned about during the pandemic. The theoretical and practical implications are discussed.

## Introduction

With the recent COVID-19 outbreak, people around the world are facing tremendous physical health threats as well as mental health threats. According to an early 2020 report by CNN, a federal crisis health hotline that provides counseling services for disaster distress had an 891% increase in calls in March compared to the same period a year earlier (Jackson, 2020). Because of the social distancing practice, stay-at-home orders, and fear of getting infected, people are experiencing unusual stress that is likely to influence their well-being. According to the conservation of resources (COR) theory, people have some reservoir of psychological resources, and they are motivated to protect their resources (Hobfoll and Shirom, 2001; Hobfoll, 2002). Resource loss is the main component in the stress process, and COVID-19 is likely to threaten the resources of an individual from many aspects. The pandemic is likely to act as a stressor and decrease the well-being of people (Hobfoll, 2002; Bliese et al., 2017). Well-being is defined as the overall evaluation of life experienced by an individual (Diener, 1984). In this research, we focused on positive affect and negative affect as was suggested by previous studies (Diener, 1984; Kampf et al., 2020; Kramer et al., 2021).

Given the distinctive experience of the pandemic, the factors that protect people from the stress of COVID-19 might be different from what we know from the current theory of stress and well-being (Evans et al., 1987; Parkes, 1994; Ross and Mirowsky, 2002; Kammeyer-Mueller et al., 2009). The experience of the pandemic is atypical to other types of stress (e.g., work stress, family stress, and life event stress). Several studies examined the stress factors during COVID-19. Elbay et al. (2020) found that being married and having a child contributes to lower stress levels among physicians. Higher resilience scores were found to be associated with fewer COVID-19 related worries (Barzilay et al., 2020). In a study of 1,086 participants from the United States, the most reported strategies to manage stress were distraction, active coping, and seeking emotional social support (Park et al., 2020). More empirical studies are needed in this realm to explore what other factors protect people from the stress of a pandemic. Moreover, as studies suggest that the affect of the individuals and their reported well-being fluctuate from time to time (Kuppens et al., 2010; Sun et al., 2020), researchers are calling for more intensive longitudinal measurements to account for time (Kozlowski, 2015). It was suggested that empirical studies should measure time-sensitive variables from text messages, diaries, and even blogs and vlogs, which would help capture time-sensitive psychological construct (Hill et al., 2013).

The big data method is an ideal unobtrusive intensive longitudinal measurement to the understanding of the effect of a global disaster event (e.g., pandemic) on the well-being of people and it is helpful in obtaining a nationwide and diverse sample. Therefore, the purpose of this research is to utilize big data methods to capture the well-being of people, protective factors, and risk factors nationwide. The theoretical perspective and empirical findings offer significant contributions to knowledge about stress and well-being during the pandemic. First, we theorize the similarities and differences between pandemic stress and typical stressors and provide empirical evidence to support our theory. The findings have practical implications for working adults and policymakers to make better decisions in coping with pandemics. Second, the research will extend the understanding of stress and well-being by exploring the impact of COVID-19 on the well-being of working adults. COVID-19 experience is unique in several ways, and we extend the COR theory by exploring the protective factors that are uncommon in stress-coping but effective in certain situations (i.e., during the pandemic). Third, we introduce novel methods to the research of stress and well-being. The measurements of well-being are traditionally self-reported and cross-sectional, and researchers found that affect fluctuates around a baseline (Kuppens et al., 2010), and therefore, the self-reported one-time measurement is insufficient in capturing the well-being of people. We respond to the call of Kozlowski (2015) for more intensive longitudinal measurements of time-sensitive variables (e.g., affect, social connectedness) by collecting data for 24 consecutive days. In this study, we specifically focused on working adults. We did not consider other groups (e.g., students and seniors) in the research because, compared with working adults, they may have different experiences and psychological processes during the pandemic.

## Machine Learning Methods in Capturing Psychological Concepts

Traditionally, well-being variables are measured cross-sectionally or multiple times using self-report surveys (Tourangeau and Rasinski, 1988), but changes are needed. According to affective events theory (AET), within-person variances exist in self-report affect (Weiss and Cropanzano, 1996; Morgeson et al., 2015). Reports of psychological constructs are influenced by daily events and therefore are not static (Morgeson et al., 2015). In support of this view, various studies found that affect and attitudes fluctuate moment by moment (Ilies and Judge, 2002; Gabriel and Diefendorff, 2015) and across time (Boswell et al., 2009; Kuppens et al., 2010; Benedetti et al., 2015; Ritter et al., 2016; Dobrow Riza et al., 2018). Big data from social media, given proper processing, can measure affect unobtrusively and intensively. It is unobtrusive in the sense that, unlike surveys where researchers essentially stop particular individuals to answer a certain question, social media big data flows in naturally as people engage with each other and researchers mostly play a role of observing and recording the behavior of the people. It is intensive in the sense that social media big data flows in around the clock. Careful researchers can easily achieve a sustained study and modeling of an individual for weeks or even months (Wang et al., 2016). The modeling of individual personalities and well-being illustrates this point well. With well-established methods for modeling personalities and sentiment, we passively collected and aggregateed all tweets of the subject during the pandemic to reach a consistent estimate. Considering each tweet as a survey, metaphorically, we essentially surveyed our subjects around the clock (Park et al., 2015). Following the law of large numbers, by taking an average, the results better reflect the characteristics of people than any ordinary survey that captures only a particular moment (Devore, 2015).

There are primarily two approaches to taking social media data, as psychological data and coding them into psychological variables. One such approach is machine learning, which includes methods such as regression and Latent Dirichlet Allocation (Schwartz et al., 2013; Park et al., 2015), support vector machine (Hutto and Gilbert, 2015), long short-term memory, convolutional neural network (Wang et al., 2017; Husseini Orabi et al., 2018), cross autoencoder (Lin et al., 2014), and the more recently introduced transformer-based pre-trained language models (Zhang et al., 2020). The other is rule-based modeling, which includes the widely used Linguistic Inquiry Word Count (LIWC) and the sentiment analysis tool VADER (i.e., Valence Aware Dictionary and sEntiment Reasoner). LIWC is based on polarity and assigns about 4,500 words into 76 different psycholinguistic variables. VADER builds on top of LIWC and includes a much larger corpus with around 7,500 words.^1^ In addition, VADER develops valence for its corpus such that each word in the corpus carries not only a polarity but also an intensity. In this study, we use both machine-learning-based models and rule-based models. The methods have been tested thoroughly and are well established in the psychology community (e.g., LIWC) and computer science community (e.g., VADER and personality estimation).

## Theory and Hypotheses Development

### The Influence of COVID-19 on Well-Being

COVID-19 is similar to other stressors, in that it brings about cognitive, physical, and emotional stress, and is likely to decrease the well-being of people. It is evident that COVID-19 has dramatically changed the lifestyles of people and has induced a multitude of stress into the lives of working adults. We theorize that COVID-19 is likely to decrease the well-being of people because it stresses them out emotionally, cognitively, and physically (de Jonge and Dormann, 2006; Bliese et al., 2017). First of all, the more severe the pandemic is, the higher the emotional stress there would be. When there are more diagnosed cases in the area of an individual, there is a higher possibility that a person will get infected, and therefore, the strain is higher. If an area is highly infected, people may fear going out in their free time and cannot get relaxed. Breaks, relaxation, socialization activities, and vacations are necessary for people to get detached from work, generate positive emotions, and recover from work (Fritz and Sonnentag, 2006; Trougakos et al., 2008; Kim et al., 2018). However, COVID-19 and stay-at-home orders have rendered these activities impossible. When people cannot get rested and recover from work, they are more likely to feel depressed and emotionally exhausted (Dunford et al., 2012; Bliese et al., 2017). When people stay at home for a long time, they are also likely to generate negative emotions and experience “cabin fever” (Clair et al., 2021). From a cognitive perspective, people make a large number of decisions every day. The pandemic adds more uncertainty into life and may increase cognitive loading for working adults. People who work from home need to adjust to a new work style and learn to work through new technologies (e.g., Zoom and Webex). Leaders and executives also need to make decisions in the uncertain environment of the pandemic. From a physical perspective, the pandemic may increase the physical burden. People need to frequently wash their hands. They need to protect themselves when they go out and disinfect themselves after they come back home. Accordingly, the more severe the pandemic is, the more stress people are likely to experience. It will negatively influence the well-being of people and make people experience more negative emotions and less positive emotions.

*Hypothesis* 1a: Pandemic severity in an area is positively related to working adults’ negative emotions.

*Hypothesis* 1b: Pandemic severity in an area is negatively related to working adults’ positive emotions.

### Protective Factors During the Pandemic

COVID-19 is dissimilar to other stressors, in that people need to keep social distance, which changes lifestyles and brings about many experiences that are not typical in our life (Centers for Disease Control and Prevention, 2020). COVID-19 is a type of virus that is highly contagious from person to person, and therefore, one primary measure that many countries adopt to slow its spread is social distancing (World Health Organization, 2020). According to Centers for Disease Control and Prevention (2020) guidelines, people are recommended to stay 6 feet apart from each other, not gather in groups, and stay out of crowded places. Moreover, many countries and many states in the United States introduced stay-at-home order (Mervosh et al., 2020). The social distancing rules and stay-at-home orders bring major life changes in many aspects for millions of people. First of all, people cannot interact in person with friends and colleagues, who they usually interact with. Second, families and couples are forced to stay together for a long time, and conflicts (e.g., work-family conflicts, relationship conflicts) are likely to arise. Third, it forces many working adults to work from home, which is likely to blur the boundary between work and life and introduce more problems. Previous research suggests that the relationship between stress and well-being depends on a series of individual attributes and environmental factors (Parkes, 1990; Bliese et al., 2017). Individual differences such as demographics (Ross and Mirowsky, 2002), personality (Evans et al., 1987; Parkes, 1994), and self-esteem (Kammeyer-Mueller et al., 2009) influence how people perceive strain in stressful situations. However, the life experiences during the pandemic are unprecedented, which may make some factors that are not so important in common stress coping particularly important during the pandemic time. Based on the above rationale, we propose that some factors might serve as protective factors to protect people from the psychological impact of COVID-19, and some other factors may serve as stressors that will combine with COVID-19 to strengthen the negative impact.

Based on the Big Five personality theory, we propose that working adults who are conscientious, open to new experiences, and agreeable might be more resistant to the mental impact of COVID-19. First of all, conscientiousness reflects the extent to which people are responsible and organized (Barrick and Mount, 1991). Previous research found that conscientiousness is a personal characteristic resource for stress coping because they will plan and prevent themselves from maladaptive coping (Vollrath and Torgersen, 2000). Therefore, conscientious people might be more planful in coping with COVID-19 and, hence, less negatively impacted. Moreover, conscientious people are usually more engaged in work (Barrick and Mount, 1991), and they can immerse themselves in work and other meaningful activities. Therefore, they might be more detached from the number of newly diagnosed cases, increased death toll, and other negative information from the media and, consequently, experience less stress from the pandemic.

Second, people who are high in openness may also be less impacted by COVID-19. Openness reflects the extent to which people are willing to accept new experiences (Barrick and Mount, 1991). People, who have low levels of openness, are conventional and they dislike changes and resist new experiences (McCrae and John, 1992). Previous research suggests that openness is not a strong protective factor for people to cope with stress (Vollrath and Torgersen, 2000). However, we propose that openness might be a strong personal characteristic resource for coping with COVID-19 stress. The pandemic has brought dramatic life changes to people, and they are forced to change their life routines. COVID-19 rendered many activities impossible, and people need to adapt to life changes by exploring new ways to do things and finding new activities to re-enrich their life. Therefore, people who are high in openness might be more open to the life changes that COVID-19 brings about and, therefore, mighht be more resistant to the mental threat of the pandemic.

Moreover, people who are high in agreeableness may also be less impacted by COVID-19. Agreeableness reflects the degree of friendliness, social conformity, and compliance (Barrick and Mount, 1991). During the pandemic, people are forced to stay with their partners or families for a long time and relationship conflicts are likely to arise. According to a survey of 1,200 couples, 40% of couples reported that, as a result of social distancing, they spent more than 20 extra hours per week together; only 18% of them were satisfied with their communication (Sternlicht, 2020). People who are low in agreeableness have little interest in the problems of others and do not care how other people feel (McCrae and John, 1992); they might have more relationship conflicts with others during quarantine life and, therefore, are more psychologically impacted by COVID-19. Therefore, we argue that agreeableness might be a personal characteristic resource for people to be resistant to the psychological impact of the pandemic. Accordingly, we have the following hypotheses:

*Hypothesis* 2a: Conscientiousness moderates the relationship between pandemic severity and working adults’ well-being; the negative impact is weaker for individuals who have higher levels of conscientiousness.

*Hypothesis* 2b: Agreeableness moderates the relationship between pandemic severity and working adults’ well-being; the negative impact is weaker for individuals who have higher levels of agreeableness.

*Hypothesis* 2c: Openness moderates the relationship between pandemic severity and working adults’ well-being; the negative impact is weaker for individuals who have higher levels of openness.

The COR theory states that people have some reservoir of psychological resources, which are a combination of capacities for fulfilling central needs (Hobfoll and Shirom, 2001; Hobfoll, 2002). As human beings, we need to experience a sense of belongingness and attachment to others (Ryan and Deci, 2017). Because of the stay-at-home order and social distancing practice during the pandemic, the need for people for connection might be undermined. We, therefore, propose that social connectedness should be an important resource during the COVID-19 period. In this context, social connectedness is defined as the extent to which people are connected to their social relationships (Ashida and Heaney, 2008). Connecting to families and friends helps people feel socially and emotionally connected, which is a strong protector against mental health consequences of life stress (Cobb, 1976; Ashida and Heaney, 2008). Moreover, connecting to family and friends makes social support more available, which should also make people more resistant to the psychological impact of COVID-19. Accordingly, we have the following hypothesis.

*Hypothesis* 3: Social connectedness moderates the relationship between pandemic severity and working adults’ well-being.

### Other Stressors

Resource loss is the main component in the stress process, and therefore, people are motivated to protect their resources, obtain new resources, and minimize resource loss (Hobfoll, 1989). When other stressors are combined with COVID-19 and further threaten the resources of an individual, people are more likely to have a decreased level of well-being. We, therefore, propose that age and having kids are two stressors that might strengthen the psychological impact of COVID-19.

Demographics also influence the extent to which people are sensitive to stress (Mirowsky and Ross, 1995; Ross and Mirowsky, 2002). First of all, we suggest that the pandemic is likely to stress older people more than younger people because older people are “at higher risk for developing more serious complications from COVID-19 illness” (Centers for Disease Control and Prevention, 2020). Accordingly, we have the following hypotheses:

*Hypothesis* 4: Age moderates the relationship between pandemic severity and working adults’ well-being; the negative impact is stronger as age increases.

**Figure 1:**
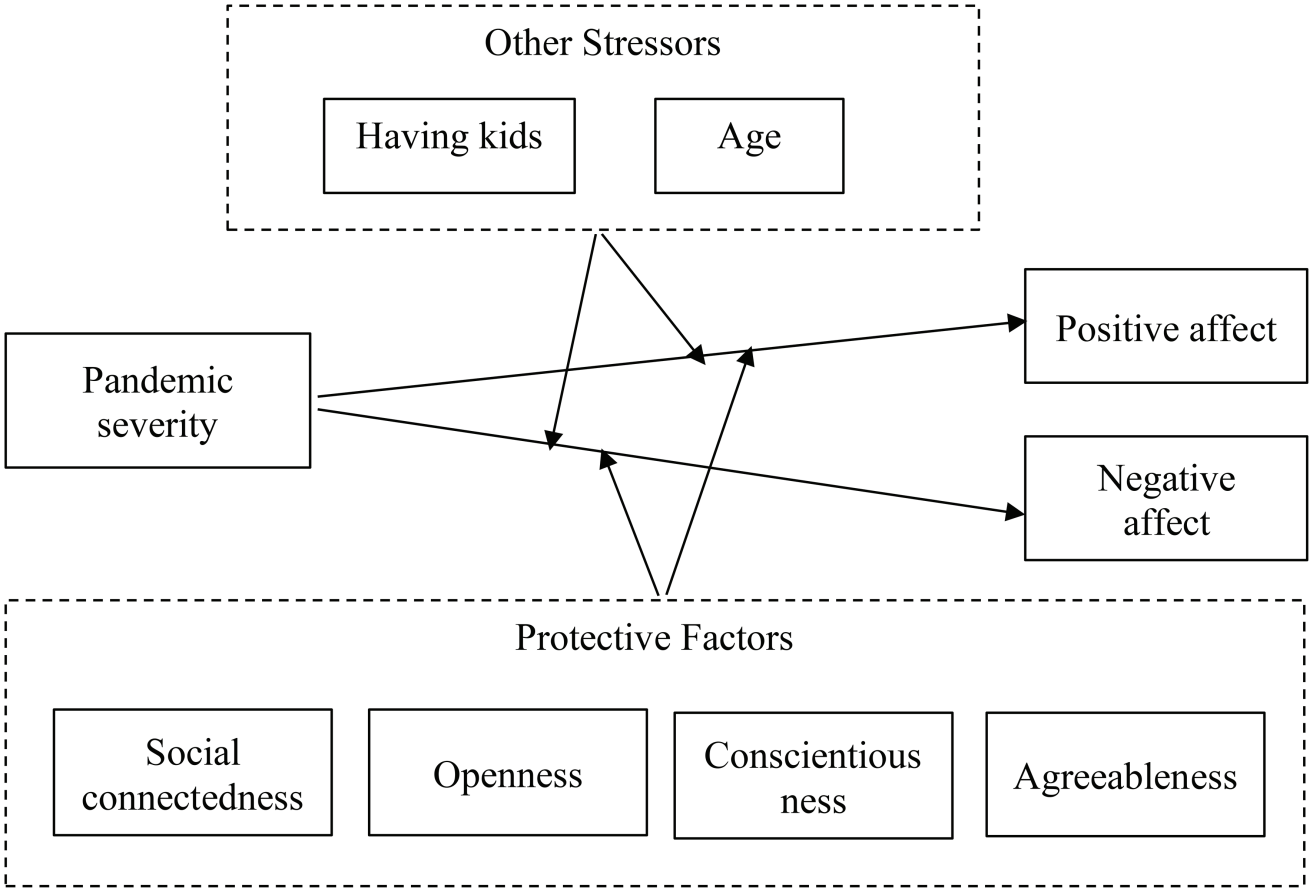
Hypothesized model.

During the pandemic, some people also experience increased levels of family responsibility, which is especially true for people with kids. For working parents, COVID-19 may increase their housework demands greatly because they need to take care of children and provide meals for them, which normally are the job of daycare centers or schools. Meanwhile, this increased level of work-family conflict also serves as an extra stressor for them. Therefore, working adults who have kids may face even more challenging situations than people with no kids. Accordingly, we have the following hypothesis, a question, and the overall model is shown in Figure 1.

*Research Question: Does having kids moderates the relationship between pandemic severity and working adults’ well-being?*

### Method

To increase the variance of pandemic severity, we need to obtain a sample where people are from as many locations as possible. We adopted a social media approach (i.e., Twitter), and we obtained a random sample of 2,231 working adults who are from 454 counties across 48 states in the US. Specifically, we used Twitter API to collect tweets posted between April 1, 2020 and April 24, 2020 with a maximum capacity of 200 tweets per user. As was discussed earlier, this study focuses on working adults, which is one of the largest groups of the whole population. The psychological impact of COVID-19 on other groups of people, despite the importance, is not of our main interests. To identify working adults, we developed an intuitive coding scheme based on the Twitter profile description of users: we aggregate all profile descriptions of users, calculate the most frequent 600 words, select occupations among them, and scan through each description of the user and label it as True if it contains one of the occupations and False if it is otherwise. We identified 37 most common occupations, including a journalist, engineer, teacher, manager, lawyer, and doctor. The people we retained in our sample meet all of our selection criteria (i.e., working adults, having a profile picture, located in the United States, and posting more than five tweets during the defined period). In total, there were 348,933 tweets in the dataset.

Twitter users normally do not disclose their demographics. However, they may use a selfie or a group photo as their profile image. To obtain the demographics, we chose to use the profile image as the source of inferring demographics. We applied the computer vision API from Face++ to analyze the profile images of Twitter users to obtain the estimates of age and gender.^2^ To increase the precision of our inference method, we inferred the demographics if there was only one intelligible face in the picture. The inference for gender is binary. Non-binary gender cannot be reached using Face++. The gender and age inferred by analyzing could be a potential bias, which, however, could be alleviated with the state-of-the-art performance of Face++ on demographics inference (Jung et al., 2018) and the large sample size. The results showed that 41.4% of the sample were female and 58.6% were male. About 8.1% of the sample were between 18 and 24 years old, 22.1% of the sample were between 25 and 34 years old, 23.9% of the sample were between 35 and 44 years old, 21.9% were between 45 and 54 years old, 15.2% were between 55 and 64 years old, and 8.6% were above 65 years old.

Using the 2013 NCHS Urban-Rural Classification Scheme for Counties (2014), we classified the counties into three groups based on the population size. The results showed that 97.03% were from the metropolitan areas, 2.82% were from the micropolitan areas, and the rest were from the noncore area.

### Measurement

#### Personality

We obtained estimates of the Big Five personality characteristics from the Personality Insights of IBM,^3^ which include agreeableness, conscientiousness, extraversion, consciousness, and openness. The IBM Personality Insights service applies data analytics algorithms to extract estimates of personality characteristics from textual inputs (Gou et al., 2014; ElSherief et al., 2018). In this case, we aggregated each tweet of the user into a single textual input to estimate the personality characteristic of the user, as commonly done in the literature (e.g., Hu et al., 2016).

#### Pandemic Severity

We retrieved the locations of the users (i.e., city and state) from their profiles. We used the python *uszipcode* package to retrieve the county given the city and state.^4^ The county label was then used to map each user to the number of confirmed COVID-19 cases and deaths at the county level on April 16, 2020.^5^ In this way, we were able to compute the number of confirmed cases and confirmed deaths at the county level for everyone.

#### GDP Per Capita

The county-level GDP data comes from the Bureau of Economic Analysis. The county-level population GDP data comes from the New York Times. We divided GDP by population to calculate the GDP per capita.^6^

#### Having Kids

We identified users who showed evidence that they are either fathers or mothers. In tweets, we performed a regular expression search for patterns, including “my/our (1–20) year old,” “my/our … X,” and “I have … X” where X represents words that stand for kids, including “boy(s),” “girl(s),” “kid(s),” and “child(ren).” In descriptions, we searched for keywords indicating the role of a parent, including “mother,” “father,” “mom,” and “dad”; we excluded users whose descriptions contain “grandpa/grandfather” or “grandma/grandmother” to make sure that the set is approximately void of seniors whose children are economically self-sufficient. Following this procedure, we labeled 462 out of 2,231 (20.7%) users in the dataset as having kids and the rest as not explicitly having kids.

#### Social Connectedness

We gathered signals on social connectedness from LIWC.^7^ LIWC is a “transparent text analysis program that counts words in psychologically meaningful categories” (Tausczik and Pennebaker, 2010). In this case, we aggregated the tweets of a user and retrieved the *family* and *friend* scores of the user from LIWC. According to the development manual of LIWC, the *family* includes 118 words (e.g., daughter, dad, and aunt) that talk about family; the *friend* includes 95 words (e.g., buddy, friend, and neighbor) that talk about friends (Pennebaker et al., 2015). Higher scores in family and friend dimensions mean that someone talks about family members or friends more frequently (Pennebaker et al., 2015), making them good proxies for social connectedness.

#### Well-Being

We estimated the positive affect and negative affect of a user using the open-source sentiment analysis tool VADER. VADER is a lexicon and rule-based model developed by researchers from the Georgia Institute of Technology (Hutto and Gilbert, 2015). Similar to personality estimates, we aggregated the tweets of a user into a single text, applied VADER, and retrieved its scores for positive and negative emotions. Positive scores and negative scores represent the proportions of text that fall in each sentiment category. For example, “this book is good” has a positive score of 0.492 and a negative score of 0. “Staying at home all day makes me so sad.” has a positive score of 0 and a negative score of 0.333.^8^ Recall that the metric for pandemic severity is based on confirmed COVID-19 cases and deaths on April 16, 2020. So here, we focused exclusively on tweets posted between April 16, 2020 and April 24, 2020 as input to estimate the well-being of a person.

**Figure 2:**
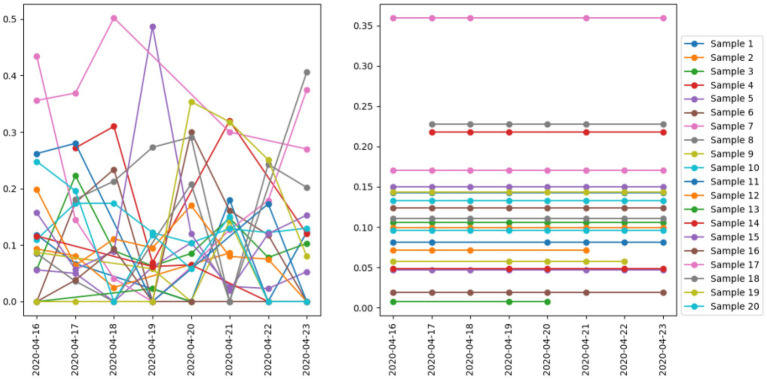
Positive affect fluctuates on a daily basis, highlighting the necessity of intensive measurement. Note. On the left, it shows the daily estimated positive affect for 20 randomly sampled users. On the right, it shows the averaged results for the same 20 users.

Recall that one of the advantages of the study is intensive measurement. In this study, we re-emphasize our point using Figure 2. On the left, we plot the positive emotion for each day between April 16, 2020 and April 23, 2020 for 20 users that we randomly sample from the dataset. Days, when a person does not post a tweet, are not plotted. One immediately notices the sizable fluctuation exhibited by virtually all users, which in part reflects the daily changes in mood of an individual. Selecting any day as the measurement would cause a significant measurement error. By contrast, using intensive measurement, we observe each individual for a sustained period of time and estimate their emotion with all the data points from an entire week. By the law of large numbers, our measurement is less prone to measurement error than sampling any random day.

## Results

### Hypotheses Testing

We used SPSS 25.0 to perform descriptive analysis and regressions, and we used the SPSS PROCESS Macro (Hayes, 2018) to test the moderation hypotheses. Table 1 reports the means, SDs, and correlations of all the studied variables.

**Table 1:** Means, SDs, and correlations.

	Mean	SD	Gender	Age	Cases	Deaths	Positive affect	Negative affect	Openness	Conscientiousness	Agreeableness	Friend Connectedness	Family connectedness
Gender	0.41	0.49											
Age	43.6	14.2	−0.002										
Cases	3,317	4,758	−0.012	−0.044^∗^									
Deaths	130	209	−0.002	−0.036	0.951^∗∗∗^								
Positive affect	0.137	0.086	0.078^∗∗∗^	−0.046^∗^	0.023	0.013							
Negative affect	0.073	0.066	0.016	0.032	0.042^∗^	0.044^∗^	−0.114^∗∗∗^						
Openness	0.80	0.18	−0.170^∗∗∗^	0.126^∗∗∗^	0.029	0.016	−0.115^∗∗∗^	0.079^∗∗∗^					
Conscientiousness	0.43	0.29	0.040	0.161^∗∗∗^	−0.045^∗^	−0.057^∗∗^	0.066^∗∗^	−0.131^∗∗∗^	0.094^∗∗∗^				
Agreeableness	0.32	0.26	0.304^∗∗∗^	0.079^∗∗∗^	−0.047^∗^	−0.046^∗^	0.275^∗∗∗^	−0.137^∗∗∗^	−0.127^∗∗∗^	0.486^∗∗∗^			
Friend connectedness	0.247	0.54	−0.041	−0.052^∗^	0.029	0.022	0.115^∗∗∗^	−0.002	−0.013	−0.028	0.015		
Family connectedness	0.326	0.90	0.040	−0.026	0.013	0.002	0.109^∗∗∗^	0.030	−0.044^∗^	0.037	0.065^∗∗^	−0.001	
Kids	0.21	0.41	0.092^∗∗∗^	0.045^∗^	−0.053^∗^	−0.052^∗^	0.037	−0.022	−0.014	0.006	0.059^∗∗^	−0.011	0.058^∗∗^

*n* = 2,231; Gender: 0 = male, 1 = female; Kids: 0 = no kids, 1 = having kids.

∗ *p* < 0.05;

∗∗ *p* < 0.01;

∗∗∗ *p* < 0.001.

As shown in Table 2, after we controlled for gender and county-level GDP per capita, confirmed cases in the county significantly predicted negative affect (Model 1: *b* = 0.07, *SE* = 0.03, *p* = 0.018) but did not significantly predict positive affect (Model 3: *b* = 0.04, *SE* = 0.04, *p* = 0.29). When we used the number of deaths in the county as a predictor, deaths significantly predicted negative affect (Model 2: *b* = 1.55, *SE* = 0.67, *p* = 0.02), but similarly, deaths did not significantly predict positive affect (Model 4: *b* = 0.51, *SE* = 0.87, *p* = 0.56). Thus, hypothesis 1a was supported, but hypothesis 1b was not supported.

**Table 2:** The main effect of pandemic severity on the well-being of working adults.

	Negative affect		Positive affect	
	Model 1	Model 2	Model 3	Model 4
Intercept	8.11(0.37)^∗∗∗^	8.11(0.36)^∗∗∗^	11.82(0.47)^∗∗∗^	11.87(0.47)^∗∗∗^
Gender	0.21(0.28)	0.21(0.28)	1.36(0.37)^∗∗∗^	1.36(0.37)^∗∗∗^
GDP	−0.02(0.004)^∗∗∗^	−0.02(0.004)^∗∗∗^	0.003(0.006)	0.003(0.005)
Cases	0.07(0.03)^∗^		0.04(0.04)	
Deaths		1.55(0.67)^∗^		0.51(0.87)
Total R^2^	0.008	0.008	0.007	0.006

*n* = 2,231; values are unstandardized coefficients with SEs in parentheses; cases are re-scaled to 1,000 cases.

∗ *p* < 0.05;

∗∗∗ *p* < 0.001.

**Figure 3:**
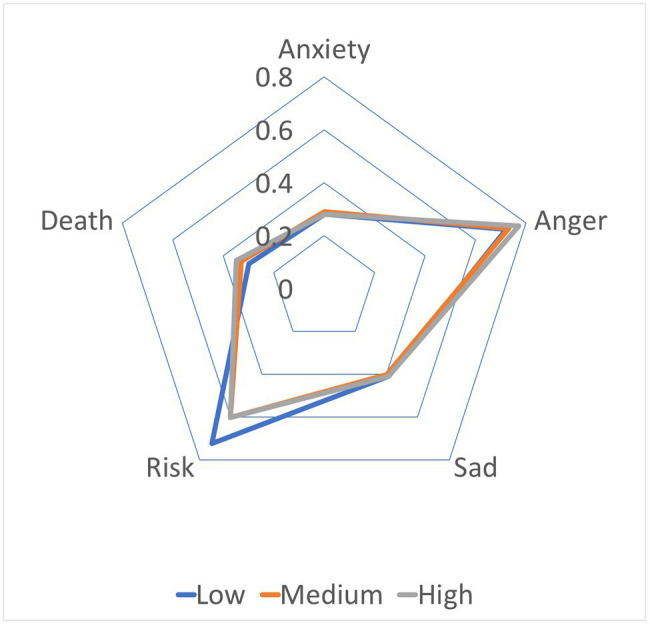
Exploration of categories of negative emotions. Note. We divided the case numbers by percentiles into three groups, low-risk group (fewer than 564 cases), medium-risk group (between 564 cases and 2,085 cases), and high-risk group (higher than 2,085 cases).

To further explore what negative emotions that pandemic led to, we dived into the LIWC analysis for these 2,231 working adults in the dataset. As shown in Figure 3, we found out that, in areas where the pandemic threat was low, people were more concerned about risk; in areas where the pandemic threat was high, people were more concerned about death and expressed more anger; in areas where the pandemic threat was moderate, people showed more anxiety.

Since the main effect of pandemic severity on negative affect was significant, we further tested the moderation effect of hypothesized moderators on this relationship. Because the correlation between confirmed cases and confirmed deaths was high (*r* = 0.951, *p* < 0.001), and the effect of cases and deaths on negative affect were similar, we only tested the moderation effects using confirmed cases representing the pandemic severity. In SPSS PROCESS Macro, we set bootstrap estimates at 5,000 times for the construction of 95% bias-corrected CIs.

We utilized Model 1 to examine the added variance of the interaction term. As shown in Table 3, the interaction term of cases and openness was significant (*b* = −0.418, *SE* = 0.153, *p* = 0.007), and the interaction term of cases and conscientiousness was marginally significant (*b* = −0.163, *SE* = 0.099, *p* = 0.10). The interaction term of cases and agreeableness (*b* = −0.044, *SE* = 0.104, *p* = 0.67), age (*b* = −0.001, *SE* = 0.002, *p* = 0.677), and having kids (*b* = −0.102, *SE* = 0.075, *p* = 0.174) were not significant. The interaction term of cases and family connectedness was also significant (*b* = −0.085, *SE* = 0.033, *p* = 0.011), but the interaction term of cases and friend connectedness (*b* = −0.081, *SE* = 0.05, *p* = 0.108) was not significant. Therefore, Hypotheses 2a and 2b were supported. Hypothesis 3 was partially supported (i.e., the family was supported while the friend was not). Hypotheses 2c and 4 were not supported. For Question 1, having kids was not a significant moderator of the relationship between the pandemic severity and well-being.

**Table 3:** Moderation effects on the relationship between confirmed cases on the negative affect of working adults (*N* = 2,231).

Hypothesis		*b*	*SE*	R^2^
2a	Gender	0.481	0.273	
	GDP	−0.017^∗∗∗^	0.004	
	Cases	0.401^∗∗^	0.127	0.020
	Openness	4.192^∗∗∗^	0.889	
	Cases × Openness	−0.418^∗∗^	0.153	
2b	Gender	0.354	0.268	
	GDP	−0.017^∗∗∗^	0.004	
	Cases	0.128^∗^	0.049	0.029
	Conscientiousness	−2.324^∗∗∗^	0.555	
	Cases × Conscientiousness	−0.163^†^	0.099	
2c	Gender	0.898^∗∗^	0.281	
	GDP	−0.018^∗∗∗^	0.004	
	Cases	0.073	0.042	0.034
	Agreeableness	−3.602^∗∗∗^	0.619	
	Case × Agreeableness	−0.044	0.104	
3	Gender	0.206	0.283	
	GDP	−0.016^∗∗∗^	0.004	
	Cases	0.091^∗∗^	0.032	0.010
	Friend connectedness	0.282	0.323	
	Cases × Friend connectedness	−0.081	0.050	
	GDP	−0.016^∗∗∗^	0.004	
	Cases	0.098^∗∗^	0.032	0.012
	Family connectedness	0.516^∗∗^	0.192	
	Cases × Family connectedness	−0.085^∗^	0.033	
4	Gender	0.217	0.283	
	GDP	−0.015^∗∗∗^	0.004	
	Cases	0.109	0.096	0.009
	Age	0.016	0.012	
	Cases × Age	−0.001	0.002	
Q	Gender	0.250	0.284	
	GDP	−0.016^∗∗∗^	0.004	
	Case	0.087^∗^	0.33	0.010
	Having kids	−0.147	0.411	
	Case × Having kids	−0.102	0.075	

Cases are rescaled to 1,000 cases.

∗ *p* < 0.05;

∗∗ *p* < 0.01;

∗∗∗ *p* < 0.001.

† *p* < 0.10.

**Figure 4:**
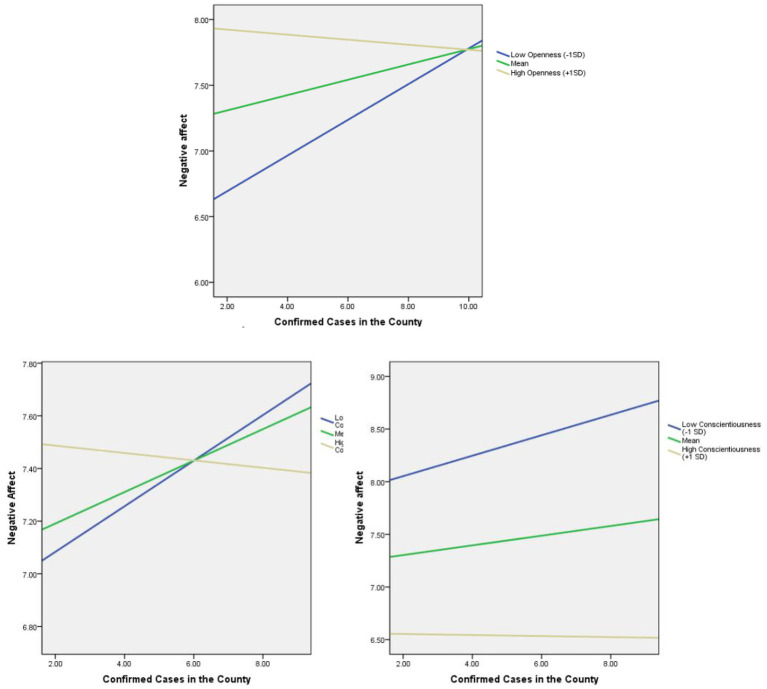
Moderating effect of openness, conscientiousness, and family connectedness on the relationship between confirmed cases and negative affect. Note. *X*-axis: confirmed cases in 1,000 s. *Y*-axis: range between 0 and 10.

As shown in Table 4, we examined a simple slope to further explore the nature of significant interactions. For each moderator, we computed the simple slopes at “high” (1 SD above the mean), “moderate,” and “low” levels of hypothesized moderators (Aiken and West, 1991). When the 95% bias-corrected CI excludes zero, there is a statistically significant effect. As illustrated in Figure 4, for openness, the influence of pandemic severity on negative affect was significant at low levels (point estimate = 0.136, 95% CI: 0.0008–0.2156) and moderate levels (point estimate = 0.058, 95% CI: 0.0035–0.1132) of openness, but the effect was not significant at high levels (point estimate = −0.019, 95% CI: −0.0947–0.0563) of openness. As illustrated in Figure 4, for conscientiousness, the influence of pandemic severity on negative affect was significant at low levels (point estimate = 0.097, 95% CI: 0.0213–0.1729) of conscientiousness, but the effect was not significant at moderate levels (point estimate = 0.046, 95% CI: −0.0086 to 0.1008) and high levels (point estimate = −0.005, 95% CI: −0.0861–0.0763) of conscientiousness. As illustrated in Figure 4, for family connectedness, the influence of pandemic severity on negative affect was significant at low levels (point estimate = 0.087, 95% CI: 0.0249–0.1483) and moderate levels (point estimate = 0.060, 95% CI: 0.0022–0.1173) of family connectedness, but the effect was not significant at high levels (point estimate = −0.014, 95% CI: −0.0951–0.0670) of family connectedness.

**Table 4:** Relationship between confirmed cases on the negative affect of working adults at different levels of hypothesized moderators (*N* = 2,231).

Moderator		Point estimate	95% Bias-corrected bootstrap confidence interval
Openness	Low (0.613)	0.136	[0.0008, 0.2156]
	Moderate (0.795)	0.058	[0.0035, 0.1132]
	High (0.978)	−0.019	[−0.0947, 0.0563]
Conscientiousness	Low (0.143)	0.097	[0.0213,0.1729]
	Moderate (0.434)	0.046	[−0.0086, 0.1008]
	High (0.724)	−0.005	[−0.0861, 0.0763]
Agreeableness	Low (0.057)	0.062	[−0.0119, 0.1368]
	Moderate (0.322)	0.047	[−0.0074, 0.1017]
	High (0.587)	0.032	[−0.0475, 0.1112]
Friend connection	Low (0)	0.080	[0.0168, 0.1433]
	Moderate (0.237)	0.061	[0.0033, 0.1187]
	High (0.777)	0.018	[−0.0590, 0.0946]
Family connection	Low (0)	0.087	[0.0249,0.1483]
	Moderate (0.33)	0.060	[0.0022, 0.1173]
	High (1.22)	−0.014	[−0.0951, 0.0670]
Age	Low (29.4)	0.078	[−0.004, 0.1605]
	Moderate (43.6)	0.061	[0.0034, 0.1187]
	High (57.8)	0.043	[−0.037, 0.1256]
Having kids	No	0.078	[0.0138,0.1419]
	Yes	−0.031	[−0.1635, 0.1017]

5,000 bootstrap samples.

**Figure 5:**
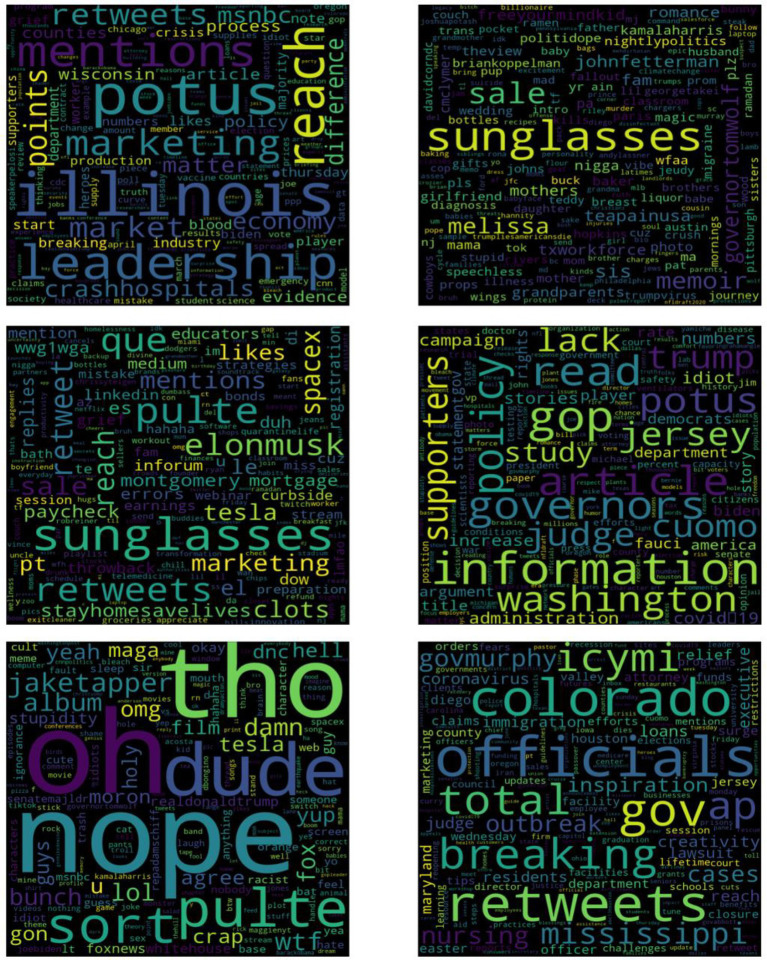
Differences in language usage between groups low in family connectedness and high in family connectedness (top), groups low in openness and high in openness (middle), and groups low in conscientiousness and high in conscientiousness (bottom). Note: The word cloud on the left shows words that are more frequently used by users low in family connectedness/openness/conscientiousness. Larger words represent a sharper contrast with users high in the construct. The word cloud on the right displays words more frequently used by those high in the construct. Larger words represent a sharper contrast with users low in family connectedness (e.g., words in the top right figure are used more frequently by people high than low in family connectedness; sunglasses are talked about 10 times more). For family connectedness, we used tweets from 1,325 users with a family connectedness score equal to 0 and 205 users whose family connectedness score was higher than 1 as per our corpus. For openness, we used tweets from 294 users with an openness score smaller than 0.6 and 775 users whose openness score is higher than 0.9 as per our corpus. For conscientiousness, we used tweets from 608 users with a conscientiousness score smaller than 0.2 and 412 users whose conscientiousness score is higher than 0.75 as per our corpus.

To further explore why these moderators (e.g., openness, conscientiousness, and family connectedness) are important during the pandemic, we generated a word cloud to explore the differences between people who are high and low in these variables. As shown in Figure 5, we found that people who are high in family connectedness talked about more things that are relaxing (e.g., sunglasses, sale, wedding, ramen, prom, vibe, photo, couch, and romance) while people who are low in family connectedness have a completely different focus (e.g., POTUS, leadership, Illinois, crash hospitals, economy, market, and industry). People who are high in openness are concerned about general politics and news (e.g., Washington, governors, Cuomo, Jersey, POTUS, policy, information, administration, American, and Fauci) while those who are low in openness are concerned about life change details (e.g., paycheck, earnings, grief, curbside, dow, mortgage, and stay home, save lives) that is likely to raise anxiety. People who are high in conscientiousness are concerned about the progress of the pandemic outbreak (e.g., total, cases, outbreak, breaking, officials, coronavirus, Colorado, and nursing) and talk more about work (e.g., clients, firm, businesses, inbox, customers, and workshop) while those who are low in conscientiousness are concerned more about entertainment (e.g., album, film, computer, game, joke, videos, and TikTok) instead of pandemic progress, and they use more emotional words (e.g., oh, nope, omg, yeal, crap, damn, and lol).

## Discussion

Using big data methods, we randomly selected people nationwide from Twitter and provided support that COVID-19 is similar to common stressors, in that it decreases the well-being of people. In general, our findings support the hypothesis that the pandemic severity relates to the well-being of working adults. Specifically, confirmed cases and confirmed deaths in the county positively influence the negative affect of working adults, but they do not significantly predict the positive affect of working adults. The results suggest that COVID-19 makes people experience more negative emotions, but it does not decrease the positive emotions of people, which is consistent with previous research that negative affect and positive affect are independent (Diener and Emmons, 1984). In this exploratory analysis, we found that, compared to those who were from low-risk counties, people in medium-risk counties experienced more anger, expressed more anxiety, and talked more about death. People in high-risk counties (more than 2,085 confirmed cases), expressed more anger and were more concerned about death than the other two groups, but they expressed less anxiety than people in medium-risk counties. Accordingly, people are likely to feel anxious, scared, and angry because of COVID-19, but the stress from COVID-19 does not prevent people from experiencing positive emotions from life. As previous research suggested that positive affect has an adaptational function during chronic stress (Folkman and Moskowitz, 2000), people during the pandemic might generate positive affect to help them adapt to life changes.

Moreover, this study also shows how COVID-19 is dissimilar to typical stressors by exploring the protective factors that play important roles during the pandemic. According to the distinctive features of pandemic experiences, we hypothesized that some factors might serve as protective factors while some other factors might act as stressors. In support of our view, we found that openness, conscientiousness, and social connectedness moderate the relationship between the pandemic severity and negative affect. Specifically, working adults who are high in openness, who are high in conscientiousness, and who are socially connected are more resistant to the negative psychological impact of COVID-19. First of all, COVID-19 is novel to everyone, and the life changes during COVID-19 are unprecedented. In this specific time, willingness to embrace change is important for people to be less psychologically impacted, making openness a characteristic resource. Working adults who are high in openness are open to new experiences (Barrick and Mount, 1991), and therefore, it is likely that they are more acceptive to the changes of life and are well adapted to the new lifestyles. The word cloud exploratory analysis was in support of our view. Specifically, we found that people who were low in openness were concerned more about life change details such as a paycheck, curbside pickup, and mortgage, while people who were high in openness cared more about politics and news that are distal to life details. It is likely that people who were high in openness were more accepting of life changes and were less bothered by life change details and, therefore, were less mentally impacted by the pandemic.

Second, the pandemic forces people to adapt to new work styles and lifestyles, making conscientiousness an important characteristic resource for working adults. Working adults who are high in conscientiousness, who are dependable, who are organized will plan ahead to prevent themselves from maladaptive coping (Vollrath and Torgersen, 2000). Therefore, conscientious people might be more planful in coping with COVID-19, and therefore, be less negatively impacted. Moreover, conscientious people are usually more engaged in work (Barrick and Mount, 1991), and they can immerse themselves in work and other meaningful activities. In support of our view, in the word cloud analysis, we found that people who were high in conscientiousness were concerned more about the progress of pandemic outbreaks and work-related issues. On the contrary, people who were low in conscientiousness were more concerned about entertainment, and they used more emotional words which made them seem unprepared and unorganized. Accordingly, during the pandemic, working adults who are high in conscientiousness closely monitor the pandemic progress, which might make them more planful and more prepared in coping with the pandemic; moreover, they can engage themselves in work or other meaningful activities, which may enable them to be less psychologically impacted by the pandemic.

Third, the pandemic, social distancing practices, and stay-at-home orders enlarge the physical distances between people and might threaten the need for connectedness among people. In support of our hypothesis, we found that social connectedness was important during the pandemic. Interestingly, the results suggest that family connectedness buffers the negative psychological impact of the pandemic while friend connectedness does not. Feeling connected to others is beneficial for well-being (Houltberg et al., 2011). During the pandemic, family connectedness could fulfill a need for relatedness, making people feel more emotionally supported and less isolated and, therefore, buffer the psychological influence of the pandemic. In the word cloud exploration, we found that people who were high in family connectedness also talked more about things that are relaxing such as sunglasses, sale, couch, and prom, which suggest that people who were family connected were able to engage in relaxing activities and therefore more resistant to the mental impact of COVID-19.

The research suggests that people may be able to adopt some measures to be more psychologically resistant to COVID-19. While personalities are usually considered to be static, people could still change personality through a self-regulation process (Hennecke et al., 2014). With that being said, people can consider the value of being more open and conscientious, and they can regulate their cognition and behaviors accordingly. Working adults can intentionally change the way they think of current situations and be more open and receptive to changes. Working adults could try to engage themselves more in work and other activities, and therefore, become more psychologically detached from the pandemic. Moreover, this research addresses the importance of family connectedness. Working adults could try to communicate with family members (e.g., parents, children, and extended family members) more frequently to increase family connectedness.

## Limitations

Despite several strengths, such as using big data methods and collecting data nationwide, this study has several limitations worth noting. First, even though we controlled for some important variables (i.e., county-level GDP per capita and gender) in the analysis, there were some other factors that we were unable to identify (e.g., ethnicity, individual-level socioeconomic status) and, therefore, left uncontrolled. For example, a CDC official report suggested that the impact of COVID-19 on people is different by race (Smith et al., 2021). Specifically, Hispanic, Black, and Indian Americans have more disproportionate COVID-19 incidents, hospitalization, and mortality than White people (Smith et al., 2021). Moreover, socioeconomic status also influences the impact of COVID-19 such that the poor are likely to experience more difficulties during COVID-19 (Raut et al., 2020). Future research may take ethnicity and socioeconomic status into consideration when studying the psychological impact of COVID-19 on people.

Second, we used novel methods in collecting our data, and some biases may exist in capturing the variables of interest. For example, for the variable “having kids,” we identified whether people have kids by scanning through their tweets and finding language patterns. We identified 21% of the people in our sample as explicitly having kids. However, there were some parents, who never mentioned their kids on Twitter, not identified as having kids in our data. Therefore, the variable of having kids might not be of high accuracy and it may explain why we did not find a significant moderation effect of having kids in the relationship between pandemic severity and the well-being of individuals. Moreover, to measure demographic variables, we only retained people who have a profile picture with an intelligible face, which might be another source of bias in our study.

## Conclusion

Using big data methods, this research investigated the mental impact of COVID-19 on 2,231 working adults nationwide by analyzing 348,933 tweets that people posted from April 1, 2020 to April 24, 2020. We found that the pandemic severity predicted the negative affect of working adults rather than the positive affect. Moreover, the relationship was moderated by personality traits (i.e., openness and conscientiousness) and social connectedness (i.e., family connectedness), which means that people who are conscientious, who are open to new experiences, and who are connected to family are more resistant to the psychological impact of COVID-19.

## Data Availability Statement

The raw data supporting the conclusions of this article will be made available by the authors, without undue reservation.

## Author Contributions

XZ and YW conceived of the presented idea and performed the statistical analyses. YW, HL, YZ, and YL collected the data. XZ took the lead in writing the manuscript. All authors provided the critical feedback and helped shape the research and manuscript.

### Conflict of Interest

The authors declare that the research was conducted in the absence of any commercial or financial relationships that could be construed as a potential conflict of interest.

## Footnotes

^1^Here words should be interpreted in the broader sense, as they include emoticons, acronyms and initialisms as well.

^2^To estimate age and gender attributes, we use the computer vision API by Face++ at https://www.faceplusplus.com/

^3^For IBM’s Personality Insight service, please see https://cloud.ibm.com/docs/personality-insights?topic=personality-insights-about#models-index

^4^uszipcode is an open-source program (https://pypi.org/project/uszipcode/).

^5^The data we used for confirmed cases and deaths was from the New York Times. For viewing and downloading the original data files, please visit https://github.com/nytimes/covid-19-data

^6^Bureau of Economic Analysis: https://www.bea.gov/data/gdp/gdp-county-metro-and-other-areas. New York Times: https://github.com/nytimes/covid-19-data

^7^To experiment with the program, please visit https://liwc.wpengine.com/

^8^For the VADER’s documentation and demo, please visit https://github.com/cjhutto/vaderSentiment
